# Evaluating criminal justice reform during COVID-19: The need for a novel sentiment analysis package

**DOI:** 10.1371/journal.pdig.0000063

**Published:** 2022-07-13

**Authors:** Divya Ramjee, Louisa H. Smith, Anhvinh Doanvo, Marie-Laure Charpignon, Alyssa McNulty-Nebel, Elle Lett, Angel N. Desai, Maimuna S. Majumder

**Affiliations:** 1 Department of Justice, Law and Criminology, School of Public Affairs, American University, Washington, District of Columbia, United States of America; 2 Roux Institute, Northeastern University, Portland, Maine, United States of America; 3 COVID-19 Dispersed Volunteer Research Network, Boston, Massachusetts, United States of America; 4 Institute for Data, Systems, and Society, Massachusetts Institute of Technology, Cambridge, Massachusetts, United States of America; 5 Department of Epidemiology and Biostatistics, School of Public Health, Texas A&M University, College Station, Texas, United States of America; 6 Computational Health Informatics Program, Boston Children’s Hospital and Harvard Medical School, Boston, Massachusetts, United States of America; 7 Perelman School of Medicine, University of Pennsylvania, 3400 Civic Center Boulevard, Philadelphia, Pennsylvania, United States of America; 8 Division of Infectious Disease, University of California Davis Health, Sacramento, California, United States of America; 9 Department of Pediatrics, Harvard Medical School, Boston, Massachusetts, United States of America; Emory University, UNITED STATES

## Abstract

The health and safety of incarcerated persons and correctional personnel have been prominent in the U.S. news media discourse during the COVID-19 pandemic. Examining changing attitudes toward the health of the incarcerated population is imperative to better assess the extent to which the general public favors criminal justice reform. However, existing natural language processing lexicons that underlie current sentiment analysis (SA) algorithms may not perform adequately on news articles related to criminal justice due to contextual complexities. News discourse during the pandemic has highlighted the need for a novel SA lexicon and algorithm (i.e., an SA package) tailored for examining public health policy in the context of the criminal justice system. We analyzed the performance of existing SA packages on a corpus of news articles at the intersection of COVID-19 and criminal justice collected from state-level outlets between January and May 2020. Our results demonstrated that sentence sentiment scores provided by three popular SA packages can differ considerably from manually-curated ratings. This dissimilarity was especially pronounced when the text was more polarized, whether negatively or positively. A randomly selected set of 1,000 manually scored sentences, and the corresponding binary document term matrices, were used to train two new sentiment prediction algorithms (i.e., linear regression and random forest regression) to verify the performance of the manually-curated ratings. By better accounting for the unique context in which incarceration-related terminologies are used in news media, both of our proposed models outperformed all existing SA packages considered for comparison. Our findings suggest that there is a need to develop a novel lexicon, and potentially an accompanying algorithm, for analysis of text related to public health within the criminal justice system, as well as criminal justice more broadly.

## Author summary

Incarceration is a social cause of disease, with currently and formerly incarcerated individuals being more likely to face vulnerabilities to disease outbreaks. The COVID-19 pandemic amplified the health-related shortcomings in the U.S. prison system, prompting the U.S. Department of Justice to consider reforming early release and home confinement measures. Public attention and news media coverage has concurrently increased, with particular attention to criminal justice reform initiatives and systemic ethnoracial inequities. Here, we attempted to characterize public sentiment towards criminal justice reform, in light of the pandemic and public health concerns, using existing tools to assessing positive, negative, and neutral sentiment. Our findings suggest these existing tools are inadequate for accurately gauging sentiment in texts at the intersection of public health and criminal justice, but also for potentially for texts related to criminal justice more broadly. Along with other approaches, properly validated tools for understanding sentiment can assist in gauging the scope of public health and reform measures for incarcerated persons and public support for or against criminal justice reform.

## Introduction

The disproportionate incarceration of ethnoracial minorities and marginalized populations represents one mechanism through which structural racism drives health inequity in the United States (U.S.) [1]. The coronavirus disease 2019 (COVID-19) pandemic provided an acute shock to the criminal justice system and adversely impacted the health of incarcerated people, as well as correctional workers and staff [2]. Vulnerabilities driven by exposure to the carceral system have been exacerbated by COVID-19, further impeding the health of communities of color.

Incarceration and detention facilities are disproportionately affected by infectious disease outbreaks [1], and the onset of COVID-19 prompted the U.S. Department of Justice to consider prisoner release and home confinement as mitigation options to control transmission in March 2020 [3]. While this policy only applied to facilities under the control of the U.S. Bureau of Prisons, states made varying decisions regarding prisoner release, perhaps in part due to public opinion and activist movements [4]. Thus, it is important to understand the role of correctional facilities in the pandemic, including support for reform measures, to better address the health implications of mass incarceration.

In recent years, the rhetoric related to criminal justice policy has increasingly emphasized reform, with the framing of “unfair” punishment and circumstances being most effective in garnering support [5]. News media outlets in particular have served not only to highlight existing public opinion, but also to help shape public perceptions based on their coverage [6]. The health and safety of incarcerated persons and correctional personnel were prominent in the U.S. media during the first year of the COVID-19 pandemic [1], highlighting the need to examine the discourse around public health policy in such contexts.

To understand and characterize public sentiment towards support for and against release of incarcerated individuals, we used existing natural language processing (NLP) lexicons and related algorithms to assess sentiment (people’s opinions, attitudes, evaluations, and emotions [7]) in news media coverage towards prisoner release and criminal justice reform over the course of the pandemic. NLP tools and techniques provide rapid means for analyzing large amounts of text and are increasingly used in social science and policy contexts [8]. Sentiment analysis (SA)—or opinion mining—is an NLP subfield that pairs sentiment lexicons, i.e. dictionaries of words and phrases with rated sentiment polarity (negative or positive), with specific algorithms that account for important syntactical and contextual features [8]. Common practical applications of SA span a wide range of fields including economics, marketing, politics, and public health [8].

To our knowledge, a SA lexicon specific to the field of criminal justice does not yet exist. This field is unique in that much of the related vocabulary is inherently negative, though the intentions and motivations of the discourse may be positive [4,6]—thus potentially skewing sentiment analysis of commentary in support of criminal justice reform (positive sentiment polarity) and against criminal justice reform (negative sentiment polarity). We therefore hypothesized that due to dual use [9] (i.e., using a system developed for a purpose separate from the one for which it was designed), existing SA packages (i.e., lexicon-algorithm pairs) would be insufficient for accurately gauging sentiment in news media coverage related to public health crises within the criminal justice system, particularly during the COVID-19 pandemic.

To test our hypothesis, we manually rated sentiment scores on a text corpus of news media articles related to COVID-19 and incarceration. Our manually-curated scores were then compared to ratings from existing SA packages for each sentence of the selected sample. Building on a training set consisting of our manual ratings as the reference outcome, we created two algorithms (based on a linear regression model and a random forest regression model) to improve on currently available SA tools that are not tailored to text at the intersection of public health and the criminal justice system.

## Results

Our experiment and analyses considered the following existing SA packages that are most frequently used in the NLP literature: SocialSent [10], VADER [7], and Stanford CoreNLP [11].

### Sentiment scoring and lexical analysis

**Fig 1:**
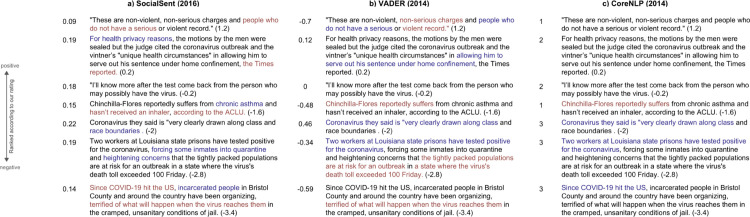
Standardized sentence sentiment scores that deviated least from manually-curated scores, exemplified by three distinct SA packages. SocialSent was considered because it is specifically attuned to social science contexts. VADER was also included for comparison because it is one of the most widely used SA packages. Finally, CoreNLP was used due to its accuracy in sentiment scoring by a recent systematic review of SA in public health. The sentences are arranged from top to bottom in order of most positive sentiment score to most negative, as determined by manual curators. The left, middle, and right panels correspond with the SocialSent, VADER, and Stanford CoreNLP SA packages, respectively. To the left of the sentences are the sentiment values assigned by each SA package on its own scale (SocialSent from 0 to 1, VADER from -1 to 1, CoreNLP 1 to 3); our ratings follow each sentence in parentheses (range of -4 to 4). Colors indicate relative sentiment associated with the selected portion of the sentence, with red and blue indicating negative and positive sentiment, respectively, as determined by running each algorithm on separate phrases within the sentences.

Overall, sentences that were manually scored to have neutral sentiment were consistently rated as neutral by the above listed SA packages. Fig 1 shows three such sentences (sentences 2–4), with scale-standardized scores (see Methods) that deviated least from our ratings, all neutral in sentiment (i.e., either fully neutral in sentiment or with equal amounts of positive and negative sentiment). However, sentences with more extreme positive or negative sentiment polarity, as ascertained by our manual ratings, were more often scored differently across SA packages (sentences 1, 5–7 in Fig 1). This division appeared driven by words related to criminal justice and public safety (e.g., “innocent”, “violent”, “defense”, “threat”, “vulnerable”, “safety”, “care”, “negative”).

**Fig 2:**
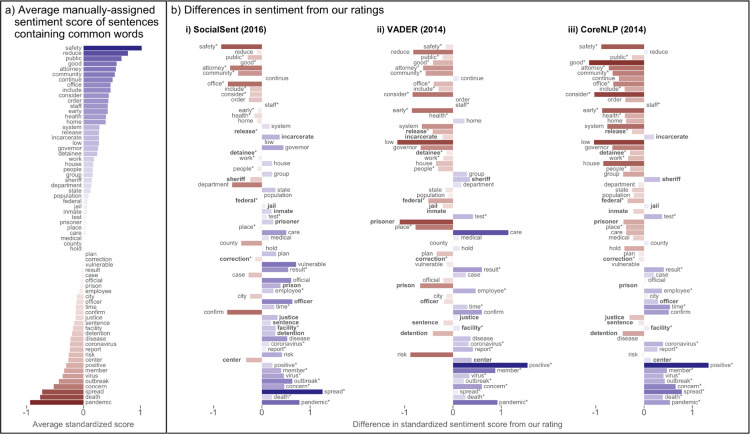
Average standardized sentiment scores for sentences containing each of the 68 most frequently appearing words. The words are arranged from top to bottom in order of most positive (blue) average sentiment to most negative (red), according to our human-curated scores (Panel A). Colors in Panel B indicate the relative score from each of three SA packages compared to our ratings, with red (blue) indicating that the algorithm-rated sentences with a given word more negatively (positively) than our team did. The color gradient indicates the intensity of the average standardized sentiment score for that term across sentences (i.e., light colors refer to terms rated more neutral, while darker colors refer to terms rated more negatively or more positively). The length of each bar depicts the average difference between a given package’s scores and ours. Words with direction of sentiment that differ from ours in each package are marked with an asterisk, and words relating to the criminal justice system are in bold.

To investigate, we used the 68 words (i.e., 3.6% of the overall corpus vocabulary) that appeared most often across all sentences (i.e., in at least 10) and compared the average sentence sentiment score for each word. This was conducted using our manually-curated sentence sentiment scores against the popular SA packages of SocialSent, VADER, and Stanford CoreNLP. Results present similar patterns across SA packages (Fig 2). Our manually-curated SA scores generally associated criminal justice and public safety terminologies (e.g., “safety”, “attorney”, “community”) with positively-scored sentences while the three existing SA packages yielded more neutral or negative sentiment ratings than our scores (e.g., an average score of 0.55 (95% CI -0.06, 1.17) for “community” compared to 0.06 (-0.36, 0.48); -0.02 (-0.62, 0.58); and -0.10 (-0.62, 0.41) from SocialSent, VADER, and Stanford CoreNLP, respectively) (full results in S1 Table). However, certain criminal justice-related terminology, including “detention”, “facility”, “sentence”, and “justice”, appeared in sentences we rated slightly more negatively in sentiment, on average, compared to the existing SA packages (e.g., an average score of -0.18 (-0.49, 0.12) for “facility” compared to 0.10 (-0.19, 0.40); -0.08 (-0.40, 0.24); and -0.04 (-0.32, 0.24) from SocialSent, VADER, and Stanford CoreNLP, respectively), though this was less consistent across packages. Criminal justice-related words associated with neutrally-scored (or equally positive and negative) sentences as determined by both existing SA packages and our manual curation included “jail”, “inmate”, “prisoner”, “medical”, and “test”. For terminology specific to public health and the pandemic (e.g., “disease”, “positive”, “virus”, “outbreak”, “spread”, “pandemic”), our manually-curated scores were primarily associated with negatively-scored sentences, with the exception of the word “health”, while the three existing SA packages rated these as more positive in sentiment compared to our scores (e.g., an average score of -0.31 (-0.46, -0.15) for “positive” compared to -0.09 (-0.44, 0.27); 1.24 (1.03, 1.45); and 1.03 (0.65, 1.41) from SocialSent, VADER, and Stanford CoreNLP, respectively).

### Proof of concept machine learning algorithms

To validate the proof of concept derived from our manually-curated sentiment analysis, we developed two machine learning (ML) algorithms–using a linear regression model and a random forest regression model–based on our sentiment ratings. After standardization of sentiment scores for the three existing SA packages and our two ML models, we trained and tested all algorithms on our text corpus. As is evidenced by the lowest mean absolute difference between our manually-curated scores and predicted sentiment scores (Table 1), both of our models strongly outperformed all three tested SA packages–signifying an important initial step in the development of a new SA package.

**Table 1:** Comparison of Model Fit Between Existing SA Packages and Our Model.

SA Model	Mean Absolute Difference in Standardized Score Prediction *(standard error)*
SocialSent	1.04 *(0*.*02)*
Stanford CoreNLP	1.03 *(0*.*02)*
VADER	0.95 *(0*.*03)*
Trained Linear Regression (binary DTM)	0.82 *(0*.*03)*
Trained Random Forest Regression (binary DTM)	0.76 *(0*.*04)*

DTM = Document Term Matrix

## Discussion

The COVID-19 pandemic instigated the U.S. Department of Justice, and specifically the U.S. Bureau of Prisons, to publicly acknowledge health-related shortcomings in the U.S. prison system and to address reforming early release and home confinement measures. Public attention and news media coverage has concurrently increased, with particular attention to criminal justice reform initiatives and systemic ethnoracial inequities [12]. This preliminary study illustrates that existing SA packages are inadequate for accurate assessment of sentiment in texts regarding such current events.

Our results suggest existing SA packages may be unable to accurately gauge sentiment in the text of news articles at the intersection of public health and criminal justice, especially in the context of the COVID-19 pandemic. VADER, one of the most widely used SA packages, scored many of the most frequently used words (Figs 1 and 2) as negative, despite our identification of the words as being neutral or positive within their respective sentence contexts. SocialSent performed better, rating these words more positively than VADER. The fact that the SocialSent SA lexicon is specifically tuned to social science contexts might explain this difference. Overall, existing SA packages performed similarly to each other, with an average error of roughly 1.0 for standardized score predictions (Table 1). However, our models’ performance demonstrates the limited utility of these packages–not only for analyzing texts that include both public health and criminal justice content, but also for texts related to criminal justice more broadly.

As suggested by our ML algorithms’ outperformance of existing SA packages, words used in texts related to public health within the criminal justice system are contextually unique. Our results demonstrate the importance of human curation as an initial step towards building a training data set that serves the development of a new SA lexicon and algorithm (i.e., package) specific to this interdisciplinary subject, as well as the importance of a new sentiment rating protocol (i.e., lexicon-algorithm pair) with texts specific to criminal justice. In future work, we aim to build off of the preliminary work presented here and develop a novel SA package tailored to texts related to public health crises within the criminal justice system, and potentially for the field of criminal justice overall.

Mass incarceration in the U.S. has been identified as an ongoing public health emergency requiring reform [13]. Sentiment analysis packages tailored to criminal justice and its public health context could be used to assess sentiment, emotions, and opinions related to the urgency of this reform. Prior studies have established the disparate impact of mass incarceration on communities of color, as well as the socioeconomic and health effects that bolster long-standing ethnoracial inequities [14–21]. Additionally, previous research has highlighted the interconnected consequences of institutional racism whereby inequities in the health and criminal justice systems can reinforce inequities in other sectors [22,23]. Incarceration is considered a “structural driver” of health inequalities, making it a social cause of disease, such that individuals currently and formerly incarcerated are more likely to face vulnerabilities to disease outbreaks and pandemics, including COVID-19 [24,25]. The existence of such interactions underscores the relevance of research on the perception and support of enhanced public health policies for incarcerated individuals.

Support for such criminal justice reform measures is a crucial step to dismantling structural racism and addressing growing health inequities. Along with other mixed methods approaches, properly validated NLP work on various text corpora, including news media, can assist in gauging the scope of public health and reform measures for incarcerated persons, public support for or against criminal justice reform related to public health, and additional factors (e.g. budgetary prioritizations, community safety, etc.) mediating reform policy decisions in response to the COVID-19 pandemic.

### Limitations & future work

There are limitations to this pilot proof-of-concept study. Firstly, because our corpus is limited in size, future work could use an expanded corpus to build a more robust model for sentiment analysis of such texts. For example, additional research could include developing a large-scale news media data aggregation process, as well as establishing a principled framework for sentence-level and article-level sentiment scoring. Secondly, as explained in our Methodology section, only 1,000 sentences were manually scored over two rounds of scoring, and doubling or tripling this number of sentences should be considered in the future to improve sample size. In addition, agreement about the “true” sentiment behind each sentence was limited, with an intraclass correlation between the scores of 0.57. However, this is comparable or better to similar measures in human-annotated texts [26–28] and to the same measure calculated on a subset of the texts used to develop the VADER algorithm (see S1 Text), reflecting inherent ambiguity and diversity of interpretation of language.

Thirdly, our methodology focused on unigram sentiment analysis; however, future investigations using bigram, trigram, or higher-level analyses may be warranted. Furthermore, varying word embedding strategies, building upon pre-existing low-dimensional vectors trained on legal and/or social science text corpora, could be tested and compared against these baselines. Lastly, as mentioned in our Methodology section, we limited our scope to only news articles published before George Floyd’s death to prevent coverage of the event from affecting our results, and future work could investigate this event more closely, particularly comparing news articles covering George Floyd’s death to news articles both before and after his death.

### Methodology

A recent systematic review [8] of SA in public health identified support vector machines and naïve Bayes classifiers as the most accurate algorithms (~70–80% accuracy) in the field, leading us to consider SA packages Stanford CoreNLP [11] and VADER [7] for our study. We also included SocialSent [10], which uses a novel algorithm to derive content-specific sentiment lexicons for texts related to social science. See S1 Text, S2 Text, and S3 Text for our code, data set, and additional SA packages examined.

### Sentiment scoring

MediaCloud [29], a searchable platform for articles from news outlets around the world, was used to collect articles related to COVID-19 and criminal justice from January 1, 2020 through May 25, 2020 at the state-level in the U.S. (see S2 Text for search query criteria). We subsequently scraped the full text of each available article. To avoid introducing event-specific coverage in our corpora of texts, we selected May 25th–the date of George Floyd’s death–as our end date. This particular event spurred an increase in news media coverage pertaining to criminal justice reform across the United States, especially as it related to excessive use of force by law enforcement [30]. Additionally, some stories about his death also discussed the topic of COVID-19 transmission during protests. Thus we limited our scope to only news articles published before George Floyd’s death, since the coverage of this event could affect our results.

We then used simple random sampling to select 1,000 sentences from our text corpus of 126,552 unique sentences for manually-curated sentiment rating in two phases. Additionally, we validated that the word frequency in this subset and the overall data set were comparable. The first 500 sentences were scored (negative, neutral, or positive) independently by five members of the research team (DR, AD, AM, MC, TC), which were used as a learning phase for the development of a standardized sentence scoring approach. Reviewers were provided with brief instructions on use of the sentiment rating scale (an integer scale from -4 (most negative) to 4 (most positive)), based on the scoring scheme used by the raters in creating VADER [7], and directed to examples from that project. All curators subsequently convened to reconcile rating discrepancies and ensure that all individuals agreed on how to score each sentence for our experiment (see S1 Text for coded data and annotation guide).

The second 500 sentences were then used for our experimental results and scored by the same five members of the research team. This set of sentiment ratings was further averaged across curators to compute the final score for each sentence. The intraclass correlation (ICC) for the second set of scores was 0.57. The ICC achieved by the team is comparable to the ICC for the NYT editorial snippet scores, which was 0.53 (see S1 Text). Such performance was deemed sufficient to proceed with our modeling efforts.

The second set of 500 sentences was additionally scored by SocialSent, VADER, and Stanford CoreNLP. All sentiment scores were then standardized (i.e., mean = 0 and standard deviation = 1 within scores from a given algorithm), and scores were compared between SA packages for each sentence. After lemmatization and removal of stop words, we then summarized sentiment related to the 68 words (3.3%) that appeared in at least 10 sentences by calculating the mean score across those sentences. We additionally assessed a selection of sentences to determine which sentiment scores from existing SA packages either deviated from or were consistent with our ratings (Fig 1). We further isolated the most frequently appearing words and compared our sentence sentiment scoring with those from SocialSent, VADER, and Stanford CoreNLP (Fig 2).

### Machine learning algorithms

We developed a proof of concept using our manually-curated scores for the first 500 sampled sentences. We compared the performance of our algorithms against the performance of SocialSent, VADER, and Stanford CoreNLP, using scores on the second set of 500 sampled sentences. All scoring systems were standardized (i.e., to have mean = 0 and standard deviation = 1) for comparison. We used binary document term matrices (DTMs) from our text corpus (i.e., a value is 1 if a word appears in a sentence; otherwise, the value is 0). 10-fold cross-validation was used to train and test a linear regression model and a random forest regression model on DTMs to predict sentiment scores (see S4 Text and S1 Fig for cross-validation explanation). We then compared the scores predicted from our models to the scores predicted from each of the SA packages (standardized to the same training data). We computed the mean absolute difference between these predicted scores in the test sets and the manually-curated scores, considered as the reference (i.e., ground truth) scores (Table 1).

## Supporting information

S1 Table Average score across sentences containing the 68 most-common words in our data. (DOCX)

S1 Text Code and Data Availability. (DOCX)

S2 Text Data Set Search Query Criteria. (DOCX)

S3 Text Robustness Check. (DOCX)

S4 Text Cross-Validation Technique. (DOCX)

S1 Fig Cross-validation technique used for our machine learning models. We randomly divided the data set into k partitions (e.g., “five-fold cross-validation” yields five partitions). The model was trained on k—1 partitions, using the last partition as the validation data set from which we made predictions and collected accuracy metrics. This train-test process was repeated k times so that every partition of the data serves as a test data set once.
(PNG)
