# Supplementary material for: Evaluating criminal justice reform during COVID-19: The need for a novel sentiment analysis package
Source: PLOS Digit Health. 2022 Jul 13;1(7):e0000063. doi: 10.1371/journal.pdig.0000063 (PMC9931240; doi:10.1371/journal.pdig.0000063)
Supplement: S1 Table — (DOCX) [file pdig.0000063.s001.docx]

*S1 Table. Average score across sentences containing the 68 most-common words in our data.*

|  | **Average standardized score (95% confidence interval) across sentences containing word** | | | |
| --- | --- | --- | --- | --- |
| **word** | *SocialSent* | *CoreNLP* | *Vader* | *Manual curation* |
| attorney | -0.09 (-0.63, 0.45) | -0.16 (-0.71, 0.40) | -0.05 (-0.56, 0.45) | 0.57 (0.06, 1.08) |
| care | 0.55 (-0.08, 1.17) | -0.33 (-0.78, 0.13) | 1.19 (0.61, 1.77) | 0.04 (-0.89, 0.97) |
| case | -0.33 (-0.65, 0.00) | 0.15 (-0.19, 0.50) | 0.22 (-0.12, 0.55) | -0.05 (-0.28, 0.19) |
| center | -0.59 (-1.04, -0.14) | -0.11 (-0.54, 0.31) | 0.13 (-0.45, 0.70) | -0.26 (-0.67, 0.15) |
| city | -0.36 (-0.89, 0.17) | -0.04 (-0.50, 0.42) | -0.26 (-0.75, 0.24) | -0.12 (-0.62, 0.38) |
| community | 0.06 (-0.36, 0.48) | -0.10 (-0.62, 0.41) | -0.02 (-0.62, 0.58) | 0.55 (-0.06, 1.17) |
| concern | -0.06 (-0.46, 0.34) | 0.14 (-0.44, 0.71) | 0.08 (-0.48, 0.64) | -0.52 (-1.19, 0.15) |
| confirm | -0.85 (-1.43, -0.28) | 0.38 (-0.16, 0.91) | 0.46 (-0.04, 0.97) | -0.14 (-0.52, 0.25) |
| consider | 0.20 (-0.34, 0.74) | -0.59 (-0.95, -0.22) | -0.39 (-1.01, 0.23) | 0.45 (0.06, 0.84) |
| continue | 0.55 (0.03, 1.07) | -0.01 (-0.52, 0.49) | 0.63 (0.15, 1.11) | 0.51 (-0.17, 1.19) |
| coronavirus | -0.09 (-0.28, 0.11) | 0.15 (-0.06, 0.37) | 0.08 (-0.14, 0.30) | -0.24 (-0.43, -0.05) |
| correction | -0.14 (-0.48, 0.20) | -0.12 (-0.42, 0.19) | -0.04 (-0.37, 0.29) | 0.00 (-0.28, 0.28) |
| county | -0.41 (-0.67, -0.15) | 0.11 (-0.19, 0.41) | -0.02 (-0.32, 0.29) | 0.02 (-0.26, 0.30) |
| death | -0.56 (-0.85, -0.27) | -0.37 (-0.66, -0.09) | -0.51 (-0.82, -0.19) | -0.76 (-1.07, -0.45) |
| department | -0.48 (-0.80, -0.16) | 0.02 (-0.36, 0.40) | 0.31 (-0.06, 0.68) | 0.14 (-0.14, 0.41) |
| detainee | 0.21 (-0.10, 0.53) | -0.05 (-0.70, 0.59) | 0.10 (-0.65, 0.86) | 0.24 (-0.45, 0.93) |
| detention | 0.08 (-0.30, 0.45) | -0.65 (-0.94, -0.35) | -0.62 (-1.08, -0.16) | -0.20 (-0.81, 0.40) |
| disease | 0.30 (-0.33, 0.94) | -0.27 (-0.92, 0.37) | 0.14 (-0.48, 0.75) | -0.23 (-0.93, 0.48) |
| early | 0.34 (-0.04, 0.72) | -0.44 (-1.02, 0.13) | -0.43 (-1.11, 0.26) | 0.43 (-0.17, 1.02) |
| employee | 0.16 (-0.28, 0.61) | 0.30 (-0.41, 1.00) | 0.40 (-0.24, 1.04) | -0.07 (-0.60, 0.45) |
| facility | 0.10 (-0.19, 0.40) | -0.08 (-0.40, 0.24) | -0.04 (-0.32, 0.24) | -0.18 (-0.49, 0.12) |
| federal | 0.06 (-0.51, 0.62) | -0.27 (-0.76, 0.21) | -0.44 (-1.02, 0.14) | 0.07 (-0.62, 0.76) |
| good | 0.54 (0.05, 1.03) | -0.56 (-0.96, -0.16) | 0.17 (-0.69, 1.02) | 0.58 (-0.04, 1.20) |
| governor | 0.70 (-0.06, 1.46) | -0.40 (-1.03, 0.23) | -0.41 (-1.12, 0.29) | 0.26 (-0.41, 0.92) |
| group | 0.37 (-0.13, 0.88) | -0.27 (-0.92, 0.37) | 0.46 (-0.20, 1.11) | 0.16 (-0.69, 1.01) |
| health | 0.24 (-0.08, 0.55) | -0.01 (-0.34, 0.33) | 0.37 (0.03, 0.70) | 0.39 (0.02, 0.76) |
| hold | 0.05 (-0.45, 0.56) | -0.40 (-0.87, 0.07) | 0.15 (-0.58, 0.87) | 0.01 (-0.67, 0.69) |
| home | 0.28 (-0.12, 0.69) | 0.02 (-0.61, 0.65) | 0.63 (0.03, 1.24) | 0.39 (-0.19, 0.97) |
| house | 0.38 (-0.12, 0.89) | -0.67 (-1.02, -0.33) | -0.18 (-0.76, 0.39) | 0.17 (-0.49, 0.83) |
| incarcerate | 0.64 (-0.04, 1.32) | 0.48 (-0.33, 1.29) | 0.06 (-0.67, 0.78) | 0.27 (-1.04, 1.59) |
| include | 0.23 (-0.16, 0.62) | 0.07 (-0.48, 0.63) | 0.18 (-0.50, 0.86) | 0.48 (0.04, 0.91) |
| inmate | 0.26 (0.09, 0.44) | -0.16 (-0.36, 0.03) | 0.03 (-0.16, 0.22) | 0.06 (-0.10, 0.22) |
| jail | 0.15 (-0.09, 0.38) | 0.16 (-0.08, 0.41) | -0.15 (-0.40, 0.10) | 0.07 (-0.19, 0.32) |
| justice | 0.18 (-0.37, 0.74) | -0.44 (-0.92, 0.03) | -0.04 (-0.64, 0.56) | -0.14 (-0.72, 0.44) |
| low | 0.29 (0.07, 0.51) | -0.77 (-1.08, -0.45) | -0.88 (-1.52, -0.24) | 0.27 (-0.20, 0.74) |
| medical | 0.16 (-0.38, 0.70) | -0.21 (-0.66, 0.24) | 0.14 (-0.35, 0.63) | 0.02 (-0.34, 0.38) |
| member | 0.06 (-0.48, 0.59) | 0.14 (-0.55, 0.82) | 0.53 (-0.03, 1.10) | -0.34 (-0.77, 0.09) |
| office | -0.23 (-0.62, 0.16) | -0.16 (-0.81, 0.49) | 0.08 (-0.41, 0.57) | 0.48 (0.07, 0.89) |
| officer | 0.50 (-0.01, 1.02) | 0.16 (-0.54, 0.86) | -0.31 (-0.94, 0.31) | -0.12 (-0.78, 0.53) |
| official | 0.56 (0.21, 0.90) | 0.00 (-0.49, 0.48) | -0.25 (-0.73, 0.22) | -0.05 (-0.48, 0.38) |
| order | 0.17 (-0.26, 0.59) | 0.05 (-0.54, 0.64) | 0.46 (-0.11, 1.02) | 0.44 (0.04, 0.83) |
| outbreak | 0.19 (-0.06, 0.45) | 0.00 (-0.49, 0.48) | -0.21 (-0.70, 0.28) | -0.44 (-1.01, 0.14) |
| pandemic | -0.16 (-0.83, 0.52) | -0.40 (-0.87, 0.07) | -0.02 (-0.79, 0.76) | -0.94 (-1.86, -0.02) |
| people | 0.07 (-0.28, 0.42) | -0.12 (-0.45, 0.20) | -0.14 (-0.44, 0.15) | 0.16 (-0.25, 0.58) |
| place | -0.01 (-0.84, 0.82) | -0.33 (-0.78, 0.13) | -0.74 (-1.40, -0.07) | 0.04 (-0.60, 0.69) |
| plan | 0.29 (-0.20, 0.79) | -0.11 (-0.63, 0.41) | -0.34 (-0.89, 0.21) | 0.00 (-0.50, 0.50) |
| population | 0.09 (-0.27, 0.45) | -0.13 (-0.57, 0.31) | -0.01 (-0.33, 0.31) | 0.08 (-0.27, 0.44) |
| positive | -0.09 (-0.44, 0.27) | 1.03 (0.65, 1.41) | 1.24 (1.03, 1.45) | -0.31 (-0.46, -0.15) |
| prison | 0.34 (0.14, 0.53) | -0.08 (-0.30, 0.14) | -0.73 (-0.90, -0.57) | -0.05 (-0.27, 0.16) |
| prisoner | 0.28 (-0.08, 0.64) | -0.39 (-0.72, -0.05) | -1.06 (-1.38, -0.75) | 0.04 (-0.33, 0.41) |
| public | 0.37 (-0.01, 0.76) | 0.42 (-0.13, 0.96) | 0.45 (0.04, 0.86) | 0.67 (0.17, 1.17) |
| reduce | 0.68 (0.11, 1.24) | 0.86 (0.13, 1.59) | -0.04 (-0.73, 0.65) | 0.78 (0.30, 1.26) |
| release | 0.26 (0.08, 0.43) | 0.02 (-0.22, 0.25) | -0.15 (-0.38, 0.08) | 0.27 (0.06, 0.49) |
| report | -0.15 (-0.56, 0.26) | 0.04 (-0.39, 0.47) | 0.17 (-0.22, 0.57) | -0.24 (-0.57, 0.09) |
| result | 0.50 (0.14, 0.87) | 0.37 (-0.43, 1.16) | 0.56 (-0.18, 1.29) | -0.04 (-0.71, 0.62) |
| risk | 0.16 (-0.17, 0.50) | -0.31 (-0.92, 0.30) | -1.14 (-1.53, -0.74) | -0.26 (-0.97, 0.46) |
| safety | 0.18 (-0.34, 0.70) | 0.14 (-0.50, 0.77) | 0.87 (0.40, 1.34) | 1.02 (0.38, 1.66) |
| sentence | 0.02 (-0.42, 0.46) | -0.28 (-0.73, 0.16) | -0.36 (-0.84, 0.13) | -0.16 (-0.62, 0.30) |
| sheriff | -0.09 (-0.70, 0.52) | 0.48 (-0.35, 1.32) | 0.50 (-0.31, 1.31) | 0.15 (-0.38, 0.67) |
| spread | 0.53 (0.04, 1.02) | 0.06 (-0.57, 0.69) | -0.62 (-1.36, 0.11) | -0.73 (-1.58, 0.12) |
| staff | 0.45 (0.19, 0.71) | 0.44 (0.02, 0.86) | 0.44 (0.02, 0.86) | 0.43 (0.02, 0.83) |
| state | 0.41 (0.12, 0.69) | -0.12 (-0.43, 0.19) | -0.03 (-0.34, 0.28) | 0.13 (-0.18, 0.43) |
| system | 0.44 (0.14, 0.74) | -0.48 (-1.01, 0.05) | -0.36 (-0.96, 0.24) | 0.28 (-0.18, 0.75) |
| test | 0.15 (-0.07, 0.37) | 0.42 (0.13, 0.72) | 0.46 (0.21, 0.72) | 0.05 (-0.13, 0.23) |
| time | 0.12 (-0.26, 0.51) | 0.41 (-0.15, 0.96) | 0.17 (-0.42, 0.75) | -0.13 (-0.78, 0.51) |
| virus | 0.09 (-0.26, 0.44) | 0.04 (-0.35, 0.43) | -0.02 (-0.40, 0.35) | -0.37 (-0.84, 0.11) |
| vulnerable | 0.68 (0.15, 1.20) | -0.05 (-0.60, 0.49) | -0.17 (-1.00, 0.66) | -0.03 (-0.75, 0.68) |
| work | 0.11 (-0.28, 0.51) | -0.14 (-0.59, 0.30) | -0.02 (-0.55, 0.52) | 0.19 (-0.32, 0.70) |
