# Supplementary material for: Evaluating criminal justice reform during COVID-19: The need for a novel sentiment analysis package
Source: PLOS Digit Health. 2022 Jul 13;1(7):e0000063. doi: 10.1371/journal.pdig.0000063 (PMC9931240; doi:10.1371/journal.pdig.0000063)
Supplement: S1 Text — (DOCX) [file pdig.0000063.s002.docx]

*S1 Text. Code and Data Availability*

All analyses were run using R version 4.0 and Python version 3.8. All scripts used for analyses, as well as text data from news media and human-curated scores on the 1000 sampled sentences and instructions to the reviewers, are available at <https://osf.io/5pmxv/>.
