# Supplementary material for: Evaluating criminal justice reform during COVID-19: The need for a novel sentiment analysis package
Source: PLOS Digit Health. 2022 Jul 13;1(7):e0000063. doi: 10.1371/journal.pdig.0000063 (PMC9931240; doi:10.1371/journal.pdig.0000063)
Supplement: S2 Text — (DOCX) [file pdig.0000063.s003.docx]

*S2 Text. Data Set Search Query Criteria*

The following search query was used to collect articles for this study: title:((coronavirus OR COVID* OR SARS) AND (jail OR prison* OR incarc* OR detain* OR offend* OR inmate* OR “correctional facility” OR “detention center”) NOT Weinstein* NOT Exotic* NOT Tekashi* NOT 6ix9ine* NOT Tiger* NOT Avenatti* NOT “R”.

Once aggregated, duplicate titles were removed if they had (1) the same date, (2) same title regardless of punctuation and capital letters, or (3) potentially different news outlets but were from the same state. Only state-level news outlets were included in this study, resulting in 2200 distinct news outlets for inclusion, with an average of 7.3 articles per outlet. Additionally, sentences that shared beginnings and/or ends of articles (often advertising statements) were manually extracted and removed from the body text across all news articles to keep core content only (117 template strings were removed; see 01_download_articles.Rmd code for more details).

For our manual curation methodology, we scored sentence sentiment on an integer scale from -4 (most negative) to 4 (most positive), using 0 for neutral. Our ratings were based on the understanding of the context of the sentences and the emotions evoked.
