# Supplementary material for: Evaluating criminal justice reform during COVID-19: The need for a novel sentiment analysis package
Source: PLOS Digit Health. 2022 Jul 13;1(7):e0000063. doi: 10.1371/journal.pdig.0000063 (PMC9931240; doi:10.1371/journal.pdig.0000063)
Supplement: S3 Text — (DOCX) [file pdig.0000063.s004.docx]

*S3 Text. Robustness Check*

For further general comparison of our proposed approach with existing SA packages, we additionally examined the Afinn lexicon [1], Liu and Hu opinion lexicon [2], and SentiWordNet [3] (results available in our code and data repository). The Afinn lexicon consists of a repository of English terms that were manually rated by Finn Årup Nielsen for valence (i.e., sentiment polarity), using an integer scoring system ranging between -5 (most negative) and +5 (most positive). The lexicon was developed based on the vocabulary used in micro-blogs and social media posts. While Afinn is better suited for analysis of sentiment in short text, we included it as a comparator for its recency, simplicity of use through R and Python wrappers, and its sizable lexicon (3300+ terms).

Additionally, the WordNet database consists of 100,000+ words that occur in varying contexts, and we included two other lexicons derived from the WordNet database for comparison: Liu and Hu opinion lexicon and SentiWordNet. The Liu and Hu opinion lexicon uses synonymy and antonymy relations iteratively, contains about 6800 terms, and was developed in 2004. SentiWordNet, developed in 2006, is a lexical resource for opinion mining that assigns each synset in the WordNet database three sentiment scores: positivity, negativity, objectivity. It was primarily designed for mining the expression of opinions in online forums and product reviews as well as longer pieces of written content, such as news articles. Additionally, it has the advantage of mapping each term with one or several scores, depending on the number of senses.

Our manually-curated models outperformed these SA packages in addition to the three main SA packages.

**S3 Text References**

1. Nielsen FÅ. A new ANEW: Evaluation of a word list for sentiment analysis in microblogs. arXiv 2011;1103.2903.
2. Hu M, Liu B. Mining and summarizing customer reviews. Proceedings of the Tenth ACM SIGKDD International Conference on Knowledge Discovery and Data Mining 2004;168-177.
3. Baccianella S, Esuli A, Sebastiani F. Sentiwordnet 3.0: An enhanced lexical resource for sentiment analysis and opinion mining. Proceedings of the Seventh International Conference on Language Resources and Evaluation 2010;2200-2204.
