# Supplementary material for: Evaluating criminal justice reform during COVID-19: The need for a novel sentiment analysis package
Source: PLOS Digit Health. 2022 Jul 13;1(7):e0000063. doi: 10.1371/journal.pdig.0000063 (PMC9931240; doi:10.1371/journal.pdig.0000063)
Supplement: S4 Text — (DOCX) [file pdig.0000063.s005.docx]

*S4 Text. Cross-Validation Technique*

The machine learning models we trained were tested via a technique known as cross-validation, which enabled us to obtain a confidence interval for the predictive accuracy of our model (see S5 Figure). In cross-validation, we randomly divided the data set into *k* partitions (e.g., “five-fold cross-validation” yields five partitions). We then trained the model on *k - 1* partitions, reserving the last partition as the validation data set from which we made predictions and collected accuracy metrics. We repeated this train-test process *k* times so that every partition of the data serves as a test data set once. The average of our accuracy metrics suggests how well our model tends to perform, while the standard deviation of these metrics indicates how these metrics might vary due to randomness in unseen data. This is because different parts of the data are used to train and test the model in each iteration.
