# Supplementary figures and images for: Evaluating criminal justice reform during COVID-19: The need for a novel sentiment analysis package
Source: PLOS Digit Health. 2022 Jul 13;1(7):e0000063. doi: 10.1371/journal.pdig.0000063 (PMC9931240; doi:10.1371/journal.pdig.0000063)

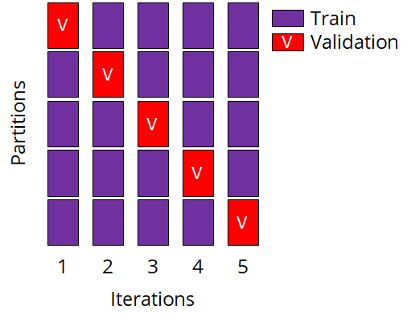

Supplement: S1 Fig — We randomly divided the data set into k partitions (e.g., “five-fold cross-validation” yields five partitions). The model was trained on k—1 partitions, using the last partition as the validation data set from which we made predictions and collected accuracy metrics. This train-test process was repeated k times so that every partition of the data serves as a test data set once. (PNG) [file pdig.0000063.s006.png]
